# Antler stem cells as a novel stem cell source for reducing liver fibrosis

**DOI:** 10.1007/s00441-019-03081-z

**Published:** 2019-08-19

**Authors:** Xiaoli Rong, Yanyan Yang, Guokun Zhang, Haiying Zhang, Chunyi Li, Yimin Wang

**Affiliations:** 1 The Scientific Research Center, China-Japan Union Hospital of Jilin University, 126 Xiantai St,, Changchun, 130033 Jilin China; 2 Department of Ultrasound, China-Japan Union Hospital of Jilin University, 126 Xiantai St., Changchun, 130033 Jilin China; 3 Institute of Special Wild Economic Animals and Plants, Chinese Academy of Agricultural Sciences, 4899 Juye St., Changchun, 130112 Jilin China; 4 Key Laboratory of Pathobiology, Ministry of Education, Norman Bethune College of Medicine, Jilin University, 126 Xinmin St., Changchun, 130021 Jilin China

**Keywords:** Antler stem cells, Liver fibrosis, HSCs, Stem cell therapy

## Abstract

Liver fibrosis results from collagen fiber deposition. Antler stem cells (ASCs) naturally in vivo differentiate into cartilage, which is only made of Col II in collagen component; whereas liver fibrosis is caused by over-abundance of Col I and III. In addition, ASCs can effectively promote regenerative wound healing in which tissue contains very few collagen fibers (Col I). In this study, we investigate the therapeutic effects of ASCs in a rat model of CCl_4_-induced liver fibrosis. Rats were treated with ASCs for 4 weeks in vivo, then biochemical and histopathological analyses were performed. Furthermore, we established cell co-culture systems of hepatic stellate cells (HSCs) and ASCs and of M1 macrophages and ASCs in vitro. Mesenchymal stem cells (MSCs) were used as a positive control. The results showed that ASC transplantation alleviated liver fibrosis effectively as evidenced by reduced collagen accumulation, decreased fatty degeneration, increased hepatocyte regeneration, decreased inflammation and significantly enhanced liver function; moreover, ASCs decreased the expression of pro-fibrogenic factors including TGF-β and α-SMA. Additionally, our study showed that ASCs inhibit HSC activation and proliferation by controlling the expression of MMPs, TIMP1, TGF-β, α-SMA and COL1A2 involved in these processes. Our results suggested that ASCs alleviate liver fibrosis effectively and inhibit HSC activation. Thus, ASCs may serve as a novel stem cell source for the treatment of liver fibrosis in the clinic.

**Electronic supplementary material:**

The online version of this article (10.1007/s00441-019-03081-z) contains supplementary material, which is available to authorized users.

## Introduction

Liver fibrosis is a pathophysiological process that refers to the abnormal increase of connective tissue in the liver caused by various pathogenic factors (Bataller and Brenner 2005). If the damaging factors are present for an extended time, the process of fibrosis will continue and develop into cirrhosis (Nishikawa and Osaki 2015). Liver transplantation is the most effective method for the treatment of end-stage liver fibrosis. However, it is limited by a lack of donor organ availability, immune rejection, surgical complications and high medical cost (Albanis and Friedman 2001). In recent years, stem cell transplantation has emerged as an effective treatment for hepatic diseases (Eom et al. 2015; Watanabe et al. 2019). Previous studies have shown that mesenchymal stem cells (MSCs) increase hepatocyte regeneration, enhance the liver functionality and reverse hepatic fibrosis (Huang et al. 2016; Kisseleva and Brenner 2012; Lan et al. 2018).

Deer antlers are the only mammalian organ that can completely regenerate every year (Li et al. 2014). According to prior studies, the annual full regeneration of deer antlers is mediated by ASCs (Li et al. 2010, Li and Suttie 2011). Compared with other stem cell sources, ASCs have the advantages of easy acquisition, high proliferative capacity and ex -vivo expansion (Li and Suttie 2011). The rationale of using ASCs to treat liver fibrosis is that ASCs naturally in vivo differentiate into cartilage, which is only made of Col II in collagen component (Li et al. 2014), whereas liver fibrosis is caused by over-abundance of Col I and III (Bataller and Brenner 2005). In addition, ASCs can effectively promote regenerative wound healing (Un-publ.), in contrast to normal scar wound healing. Scar is mainly made of Col I, whereas regenerative wound healing tissue contains very few collagen fibers (Col I) (Li et al. 2009). Consequently, we thought it worthwhile to try to use ASCs to treat liver fibrosis.

The aim of the present study is to use a rat CCl_4_-induced liver fibrosis model to investigate whether transplantation of ASCs reduces liver fibrosis in vivo. We further analyzed the molecular mechanism by which ASCs mediate their anti-fibrotic activity in vitro. Our results provided the first evidence that ASCs effectively reduce liver fibrosis. Thus, ASCs may provide a new treatment for liver fibrosis in the clinic.

## Materials and methods

### Cell culture

ASCs were obtained from a 2-year-old male sika deer (Jilin, China). Detailed procedures for primary ASC isolation and identification have been described in our previous studies (Li and Suttie 2003; Li and Suttie 2011; Sun et al. 2012). In brief, 3 cm was cut from the tip of each antler along the longitudinal axis. Then, the tip was cut into 5-mm-thick slices along the same plane. The slices were further cut into 1~2 cm strips. In order to include the full width of all tissue layers in the antler growth center, only the strips from the central area were collected. Then, these central pieces were used for primary culture. The ASCs were cultured in DMEM (Invitrogen, Shanghai, China) supplemented with 10% FBS (Gibco, Life Technologies, Australia), at 37 °C with saturated humidity and 5% CO_2_. ASCs were passaged using trypsin (Sigma, San Francisco, USA) and stored in liquid nitrogen in freezing medium (DMEM: FBS: DMSO = 6:3:1). Cells in their fifth passage were used for this study.

Wharton’s jelly-derived mesenchymal stem cells (WJ-MSCs) were generously provided by Dr. Xiuying Li (Jilin University, China). M1 macrophages were acquired from Dr. Shi in our lab. The MSCs and M1 macrophages were cultured following the same protocol as ASCs.

### CCl_4_-induced liver fibrosis in rats and cell transplantation

**Fig. 1:**
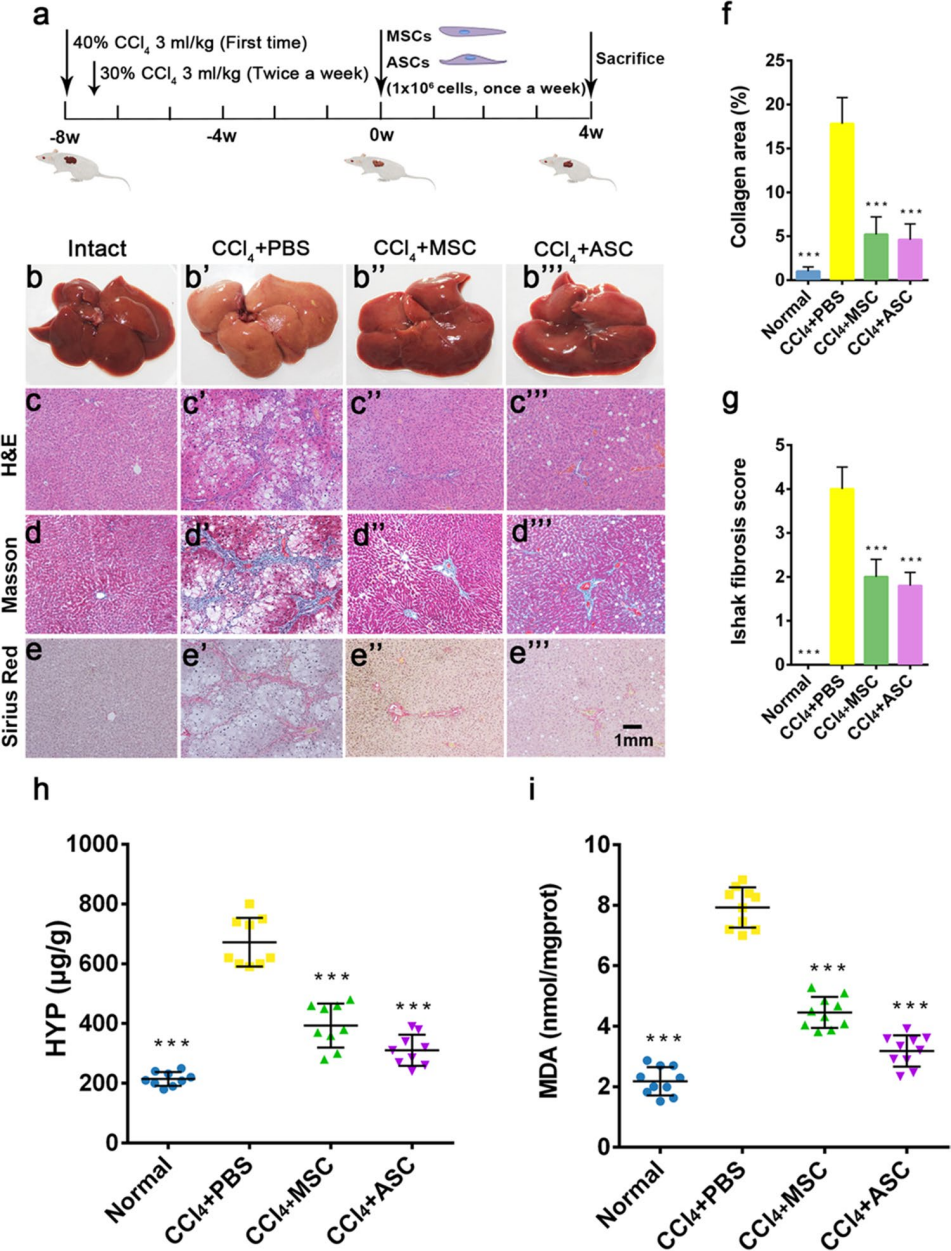
Effects of ASCs on liver fibrosis in CCl_4_ treated rats. a Experimental design. Liver (a–a″′) and liver tissue section, stained with H&E (c–c″′), Masson (b–b″′) and Sirius Red (e–e″′), bar = 1 mm. f Collagen abundance in the livers assessed by area quantification using computer-assisted image analysis. g Histopathological analysis of liver sections via Ishak scoring criteria. Ishak score from 0 to 6 (0 = no fibrosis, 6 = cirrhosis): mild (Ishak, 0–2) to severe fibrosis (Ishak, 3–6). h HYP levels. i MDA levels. Note that ASC administration significantly alleviated the liver fibrosis compared with the CCl_4_ + PBS group; no significant difference to the intact group at levels of HYP and MDA; and there was a strong trend for ASC that had a better effect on reducing liver fibrosis than MSC, although statistically not significant. ASCs: antler stem cells; MSCs: mesenchymal stem cells; HYP: hydroxyproline; MDA: malondialdehyde; mean ± SD; *n* = 10. ***p < 0.001

The animal model was made according to previously published methods with the following modifications (Cho et al. 2012). Liver fibrosis was induced in SD rats (8-week-old, female, body weight 200 g) by subcutaneous injection of CCl_4_. Forty percent of CCl_4_ was administered at an initial dose of 3 ml/kg body weight, followed by 30% CCl_4_ 3 ml/kg body weight twice a week for 8 weeks (Fig. 1a). Then, six rats were randomly selected for liver histopathological analysis to test for hepatic fibrosis formation. Next, the CCl_4_-induced rats were randomly assigned into three groups: CCl_4_ + PBS (control), CCl_4_ + MSC (positive control), CCl_4_ + ASC (treatment group), with ten rats per group (*n* = 10). Rats that had not been treated with CCl_4_ served as intact control (ten rats in intact group, *n* = 10). Rats received 1 × 10^6^/500 μl MSCs or ASCs once a week by injection through the tail vein. CCl_4_ injections were continued for a total of 12 weeks. Rats were euthanized at 4 weeks after treatment.

### Biochemical analysis

Rat blood samples were taken at 4 weeks after cell transplantation and the serum was collected. Then, alanine aminotransferase (ALT), aspartate aminotransferase (AST), alkaline phosphatase (ALP), gamma-glutamyltranspeptidase (γ-GT), direct bilirubin (DBIL), total bilirubin (TBIL), total protein (TP) and albumin (ALB) concentrations were assessed using an automated biochemical analyzer (AU-680, Beckman, Germany). Liver homogenate (10%, *w*/*v*) was prepared by homogenizing the right lobe of the liver on ice in 150 mM Tris–HCl buffered saline (pH 7.2; Sigma-Aldrich) using a polytron homogenizer (PT3100D; Kinematical, Lucerne, Switzerland). Next, the levels of hydroxyproline (HYP) and malondialdehyde (MDA) were measured using kits (NanJing JianCheng Bioengineering Institute, A030-2, A003-1, Nanjing, China) according to the manufacturer’s instructions.

### Histopathological analysis

Liver tissue sections were taken from the left lobe of the liver with 4 μm thickness. The paraffin sections were deparaffinized and rehydrated and stained with hematoxylin and eosin (H&E), Masson and Sirius red for histological examination according to the manufacturer’s standardized protocols. Briefly, Sirius Red staining was performed by incubating slides in 0.1% Sirius Red F3B for 1 h, washing twice in acidified water, dehydrating thrice in 100% ethanol and then clearing in xylene. Morphometric analysis was performed using digitally captured serial images. We used 10 random fields per section and 10 sections in total (*n* = 10 rats) for quantification of collagen deposition. The collagen-stained area was calculated via Image-Pro Plus. The degree of hepatic fibrosis was assessed according to the Ishak modified scoring system (Wu et al. 2016).

Immunohistochemistry (IHC) and immunocytochemistry (ICC) were measured with the Kit (Maixin KIT-9710, Fuzhou, China) in accordance with the manufacturer’s instructions. Briefly, the liver sections were deparaffinized, rehydrated and incubated in a 99 °C water bath for 15 min. In addition, HSCs crawled on the slide were incubated with 4% paraformaldehyde for 10 min at room temperature. Then, the slide was incubated with 3% H_2_O_2_ for 15 min and blocked with 10% normal goat serum for 1 h at 37 °C. This was followed by incubation with primary antibody against PCNA (ab15497, 1:500 dilution, Abcam, Cambridge, UK), α-SMA (ab5694, 1:500 dilution, Abcam, Cambridge, UK), TGF-β (ab92468, 1:500 dilution, Abcam, Cambridge, UK) and Col1A2 (ab96723, 1:500 dilution, Abcam, Cambridge, UK) overnight at 4 °C. Next, slides were incubated with biotinylated goat-anti-rabbit IgG antibody. We used diaminobenzidine solution as the chromogenic agent for 15 min at 37 °C, incubated with avidin peroxidase reagent and hematoxylin for counterstaining. Finally, slides were photographed using an optical microscope (Olympus, Tokyo Metropolitan, Japan). We used 10 random fields per section and 10 sections in total (*n* = 10 rats) for quantification of IHC results. The IHC results were calculated via Image-Pro Plus.

### Cell co-culture and IF staining

HSCs were resuscitated, passaged and seeded at a density of 3000 cells/cm^2^, when reaching 50% confluence. Next, 24-well plates with 0.4-μm-pore Transwell inserts were used to physically separate the two cell populations. Activated HSCs were plated in standard complete medium; SFM, MSCs and ASCs were respectively added at the same density on top of the inserts. After 48 h, the cells were incubated with 4% paraformaldehyde on 24-well plates at room temperature for 10 min, then with 1% bovine serum albumin (BSA, Biosharp, China) for 30 min. Next, cells underwent immunofluorescent labeling to detect the expression of TGF-β. Cells were incubated with primary antibody TGF-β (ab 92486, 1:100 dilution, Abcam, UK), followed by incubation with a secondary antibody (goat-anti-rabbit IgG, ab15007, 1:500 dilution, Abcam, UK) for 30 min at room temperature. F-actin was stained with rhodamine phalloidin (Thermal Scientific, USA). The nuclei were labeled with DAPI (Thermal Scientific, USA). Fluorescent images were captured by EVOS (Thermo Scientific, USA).

### Western blot

HSCs under the SFM, MSC and ASC co-culture conditions as above were cultured for 48 h followed by cell collection and protein extraction. HSC protein samples in SDS sample buffer were heated to 95 °C for 10 min and separated on SDS-polyacrylamide gels. Resolved proteins were then electro-blotted onto nitrocellulose membranes and probed with antibody against MMP1, TIMP1, α-SMA, TGF-β and GAPDH overnight at 4 °C. The antibodies were as follows: MMP1 (ab 92486, 1:1000 dilution, Abcam, UK), TIMP1 (ab 2464, 1:1000 dilution, Abcam, UK), α-SMA (ab 5694, 1:1000 dilution, Abcam, UK), TGF-β (ab 92486, 1:1000 dilution, Abcam, UK) and GAPDH (ab 8245, 1:1000 dilution, Abcam, UK). These were followed by secondary antibody HRP-conjugated goat-anti-rabbit IgG (ab15007, 1:1000 dilution, Abcam, UK) and visualized by chemiluminescent detection according to the manufacturer’s instructions (Immobilon western chemiluminescent HRP substrate, Millipore).

### Quantitative real-time PCR

HSCs under the SFM, MSCs and ASCs co-culture conditions as above were cultured for 48 h followed by HSC collection and mRNA extraction. In addition, M1 macrophages were co-cultured with SFM, MSCs and ASCs. M1 macrophages were plated in standard complete medium; MSCs and ASCs were added at the same density on top of the inserts. After 48 h of co-culture, M1 macrophages were collected and mRNA was extracted. Total RNA was isolated from the cells using Trizol reagent (Invitrogen, Shanghai, China) according to the manufacturer’s protocol. Total RNA (1 μg) was reverse-transcribed and the resulting cDNA was used as a template in qRT-PCR using a standard SYBR premix Ex Taq (Invitrogen, Shanghai, China) using the Real-Time PCR Detection System (Roche, Basel, Switzerland). GAPDH served as the internal control and experiments were conducted in triplicate. The primers are listed in Table 1. All reactions were performed in triplicate and the data were analyzed using the 2^−ΔΔCt^ method.

**Table 1:** Primers used for qRT-PCR

Gene name	Primers	Sequences	Product size (bp)
MMP1	Forward	agacagccgcatcttcttgt	156
	Reverse	cttgccgtgggtagagtcat	
MMP2	Forward	atgacagctgcaccactgag	174
	Reverse	atttgttgcccaggaaagtg	
MMP8	Forward	gaagacgcttccatttctgc	152
	Reverse	ttgcatcagtgcagttcctc	
MMP9	Forward	ggtaatgctgagggtgcaat	217
	Reverse	caaaaatgaaggggaaagca	
MMP13	Forward	ttgagctggactcattgtcg	168
	Reverse	tcacctctaagccggagaaa	
TIMP1	Forward	tcagattatgccagggaacc	175
	Reverse	cttgccgtgggtagagtcat	
zα-SMA	Forward	actgggacgacatggaaaag	195
	Reverse	tacatggcagggacattgaa	
TGF-β	Forward	atacgcctgagtggctgtct	164
	Reverse	tgggactgatcccattgatt	
IL-1	Forward	atttccgccttccagagaat	186
	Reverse	gagtctcatgggggaattga	
IL-2	Forward	aaactccccatgatgctcac	172
	Reverse	gaaatttccagcgtcttcca	
IL-6	Forward	ccggagaggagacttcacag	195
	Reverse	acagtgcatcatcgctgttc	
TNF-α	Forward	gtgacgtggagttgggtctt	187
	Reverse	gagtccgtcttggtcagagc	
GAPDH	Forward	agacagccgcatcttcttgt	158
	Reverse	cttgccgtgggtagagtcat	

### Statistical analysis

Statistical analysis was performed using Prism 6 (Graph Pad software). Multiple comparisons were analyzed by one-way ANOVA, followed by post hoc Tukey test. All quantitative data were given as the mean ± SD for at least three independent experiments. Differences were considered significant at *p* < 0.05.

## Results

### Effects of ASCs on liver fibrosis in CCl_4_ rats

To confirm the effects of ASCs on liver fibrosis, we used a CCl_4_-induced liver fibrosis model in vivo (Fig. 1a). Rats were injected with CCl_4_ for 8 weeks to induce liver fibrosis, which was then treated with MSCs (CCl_4_ + MSC) and ASCs (CCl_4_ + ASC) for 4 weeks. The sham treated with PBS (CCl_4_ + PBS) is the control. In comparison to intact rats (Fig. 1b), the livers of the CCl_4_ + PBS-treated group (Fig. 1b′) were enlarged, coarser and nodular on the surface and liver lobes were fused with each other and to the peritoneal organs. The histopathological results showed that rats treated with ASCs (Fig. 1b″′) showed a reduction in surface coarseness, became reddish, smoother and more lustrous compared with CCl_4_ + PBS-treated (Fig. 1b′); and the collagen fiber area was reduced in the ASC-treated group (Fig. 1c″′–e″′). Moreover, we quantified the Masson-stained and Sirius red-stained areas to analyze the area of collagen fibers. The percentage of collagen area was significantly decreased in ASC-treated rats as compared to CCl_4_ + PBS-treated (Fig. 1f, *p* < 0.001). Furthermore, the mean Ishak score showed a statistically significant reduction in the ASCs-treated group as compared to CCl_4_ + PBS-treated (Fig. 1g, *p* < 0.001).

Next, we detected HYP and MDA levels that indicated the degree of change in liver collagen fibers and lipid peroxidation. The HYP and MDA levels in the liver tissue of CCl_4_ + PBS-treated rats were significantly higher than intact rats (Fig. 1h and i; *p* < 0.001). Intravenous administration of ASCs significantly reduced both HYP and MDA levels compared to CCl_4_ + PBS-treated rats. These results suggested that ASCs could alleviate CCl_4_-induced liver fibrosis and that the effects were similar to MSCs.

### Effects of ASCs on liver functionality in CCl_4_ rats

**Fig. 2:**
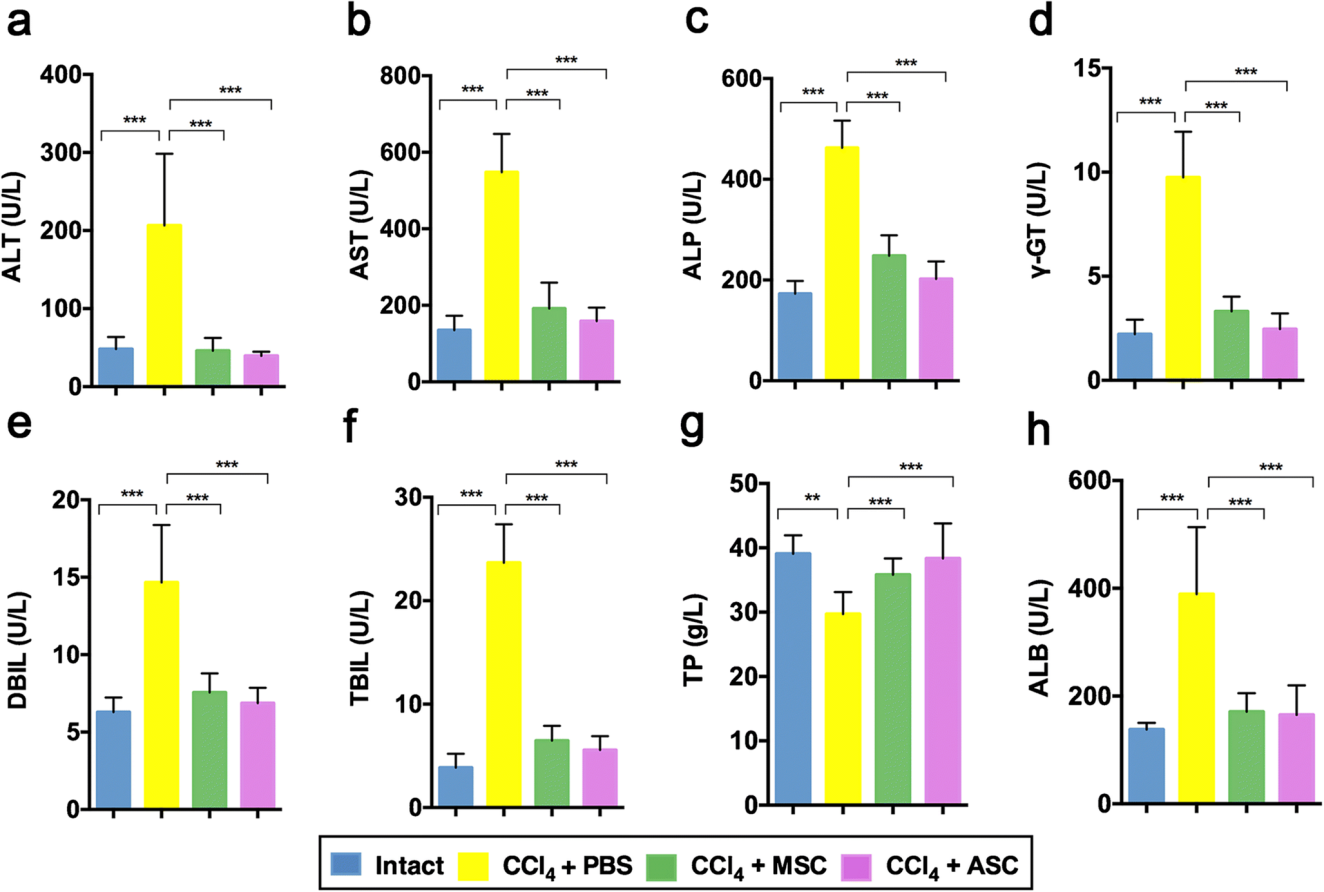
Effects of ASCs on serum biochemical parameters in CCl_4_-treated rats. **a** ALT levels. **b** AST levels. **c** ALP levels. **d** γ-GT levels. **e** DBIL levels. **f** TBIL levels. **g** TP levels. **h** ALB levels. Note that ASC administration significantly recovered liver function compared with the CCl_4_ + PBS group; CCl_4_ + PBS-treated rats significantly damaged liver function compared with the intact group; ALT: alanine aminotransferase; AST: aspartate aminotransferase; ALP: alkaline phosphatase; γ-GT: gamma glutamyl transpeptidase; DBIL: direct bilirubin; TBIL: totalbilirubin; TP: total protein; ALB: albumin; mean ± SD; *n* = 10; ** *p* < 0.01; *** *p* < 0.001

Biochemical analyses were performed to assess the restoration of liver function and decrease in liver fibrosis. In comparison to intact rats, the levels of ALT, AST, ALP, γ-GT, DBIL, TBIL, TP and ALB in CCl_4_ + PBS-treated group was significantly different (Fig. 2a–h, *p* < 0.001). The results showed that ALT, AST, ALP, γ-GT, DBIL and TBIL levels were significantly decreased in the ASC-treated group compared to the CCl_4_ + PBS group (Fig. 2a–f, *p* < 0.001). In addition, the serum level of TP in the ASCs-treated group was higher than in the CCl_4_ + PBS-treated group and ALB in the ASCs-treated group was lower than in the CCl_4_ + PBS-treated group (Fig. 2g, h; *p* < 0.001). These results suggested that ASCs improve liver function in liver injury induced by CCl_4_.

### Effect of ASCs on PCNA, α-SMA and TGF-β expression in CCl_4_ rats

**Fig. 3:**
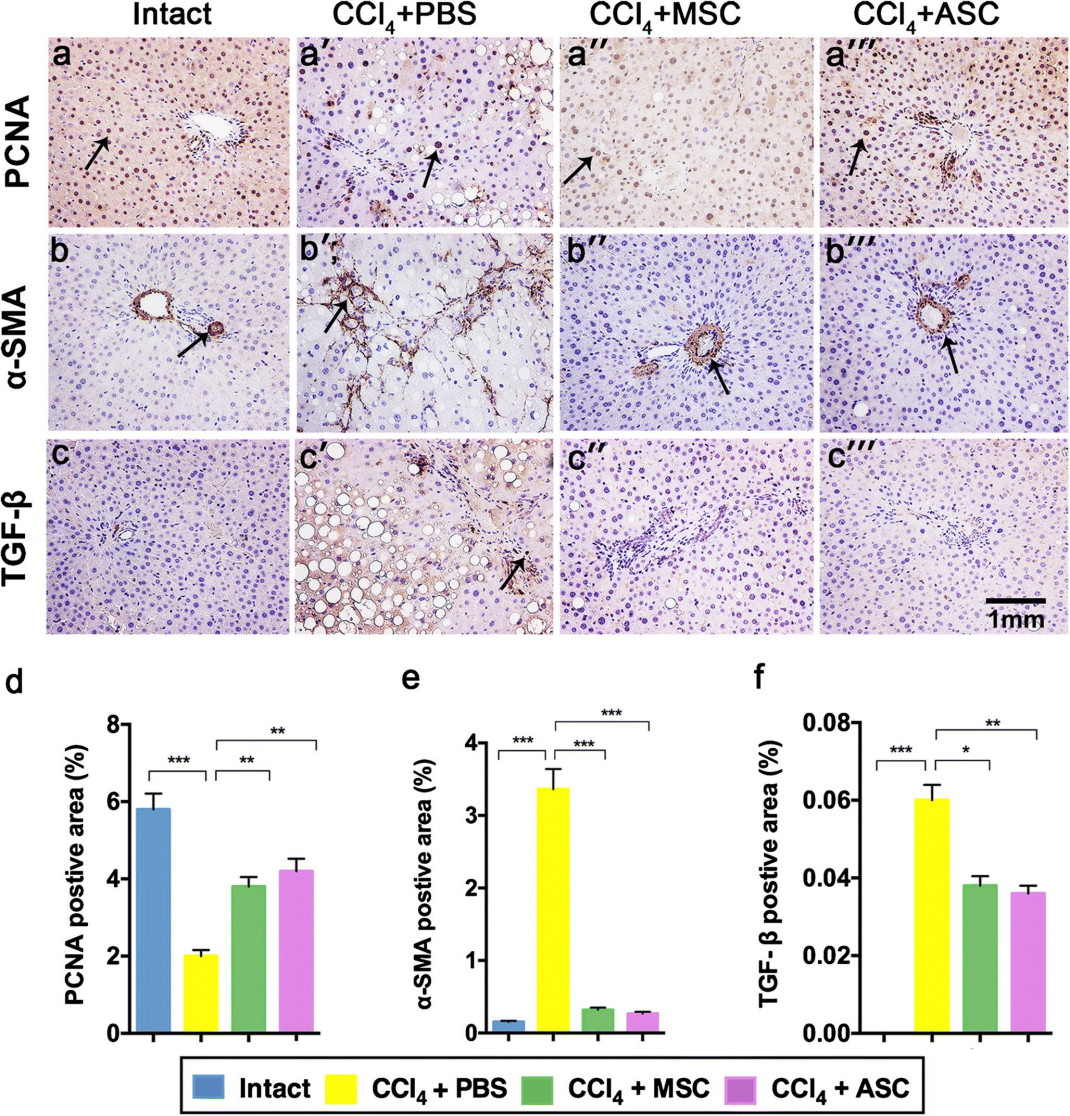
Effects of ASCs on expression of the fibrosis-related genes in CCl_4_-treated rats. Immunohistochemistry analyses of PCNA (a–a″′), α-SMA (b–b″′) and TGF-β (c–c″′) in liver tissue. d–f Positive cell area analysis of PCNA, α-SMA and TGF-β for immunohistochemical results via Image-Pro Plus. Note that ASC administration significantly increased the number of PCNA-positive cells and decreased the number of α-SMA and TGF-β-positive cells compared with the CCl_4_ + PBS group; PCNA: proliferating cell nuclear antigen; α-SMA: anti-α smooth muscle actin; TGF-β: transforming growth factor-β; mean ± SD; *n* = 10. *p < 0.05; **p < 0.01; ***p < 0.001

To determine whether transplanted ASCs promote hepatocyte proliferation, the number of PCNA^+^ cells was counted in ten random fields per rat. The results showed that the number of PCNA^+^ nuclei was significantly increased in the ASCs-treated group (Fig. 3a″′), compared to the CCl_4_ + PBS-treated group (Fig. 3a′ and d; *p* < 0.001). To evaluate the effect of ASCs on activation of HSCs, IHC was used to examine α-SMA^+^ cells in the liver sections, revealing a significantly reduced positive expression in the ASC-treated group (Fig. 3b″′) as compared to the CCl_4_ + PBS-treated group (Fig. 3b′ and e; *p* < 0.001). Similarly, we observed only a few cells with positive TGF-β^+^ expression in the intact (Fig. 3c), MSCs (Fig. 3c′), and ASC-treated groups (Fig. 3c″′), but intensely stained TGF-β^+^ cells were present around the portal and ductal region in CCl_4_ + PBS-treated liver tissue (Fig. 3c′ and 3f; *p* < 0.001). These results suggested that ASCs promoted hepatocyte proliferation and inhibited HSC activation in CCl_4_-induced liver fibrosis.

### Effect of ASC co-culture on fibrosis and inflammation-related gene expression

**Fig. 4:**
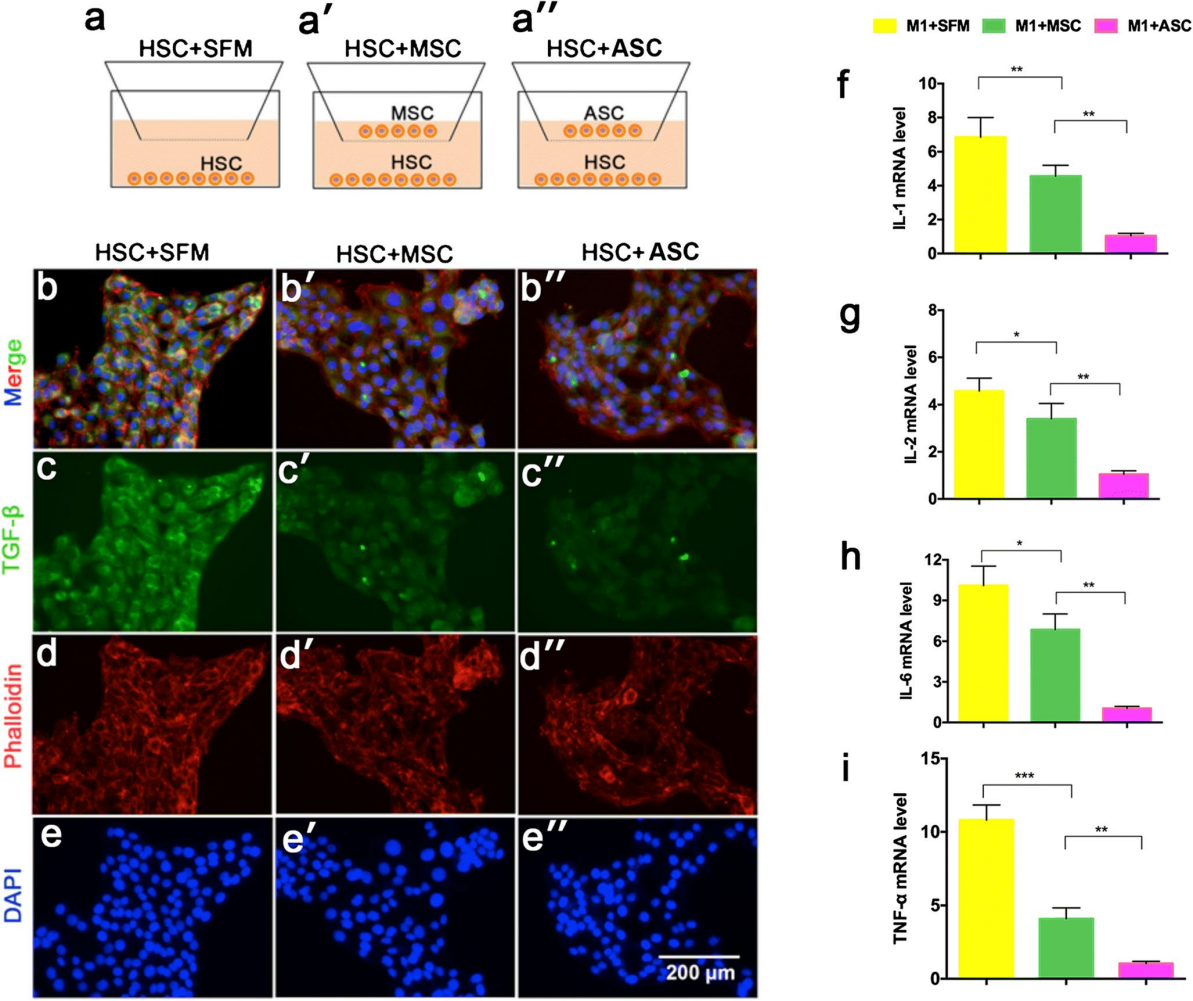
Effects of ASCs on the TGF-β in HSCs and inflammation-related gene expression in the M1 macrophage via co-culture approach. a–a″ Experimental design. Representative images of immunofluorescence staining performed for TGF-β (green, b–b″, c–c″) in HSCs. Phalloidin (red, d–d″) was stained for cytoskeleton. DAPI (blue, e–e″) was stained for nuclei. Bar = 200 μm. f–i Inflammation-related gene (IL-1, IL-2, IL-6 and TNF-α) expression in the M1 macrophage. Note that ASC administration decreased the TGF-β and inflammation-related genes expression; administration of ASC had a better effect on reducing inflammation than MSC; SFM: serum free medium; ASCs: antler stem cells; MSCs: mesenchymal stem cells; HSCs: hepatic stellate cells; mean ± SD; *n* = 3; *p < 0.05; **p < 0.01; ***p < 0.001

We detected the expression of related genes and proteins in activated HSCs under the co-culture with ASCs (Fig. 4a″). Immunofluorescence (IF) results demonstrated that the TGF-β^+^ expression was decreased in HSCs under ASC co-culture as compared to SFM conditions (Fig. 4b and b″). The results suggested that ASCs block HSCs activation via inhibiting TGF-β expression. In addition, we investigated M1 macrophage inflammatory gene expression in the different co-culture groups. The results showed that IL-1, IL-2, IL-6 and TNF-α expression was significantly decreased in the ASC-treated group, compared to the other two control groups (Fig. 4f–i; *p* < 0.05). These results suggested that ASCs decreased the TGF-β and inflammation-related gene expression; moreover, ASC had a better effect on reducing inflammation than MSC.

**Fig. 5:**
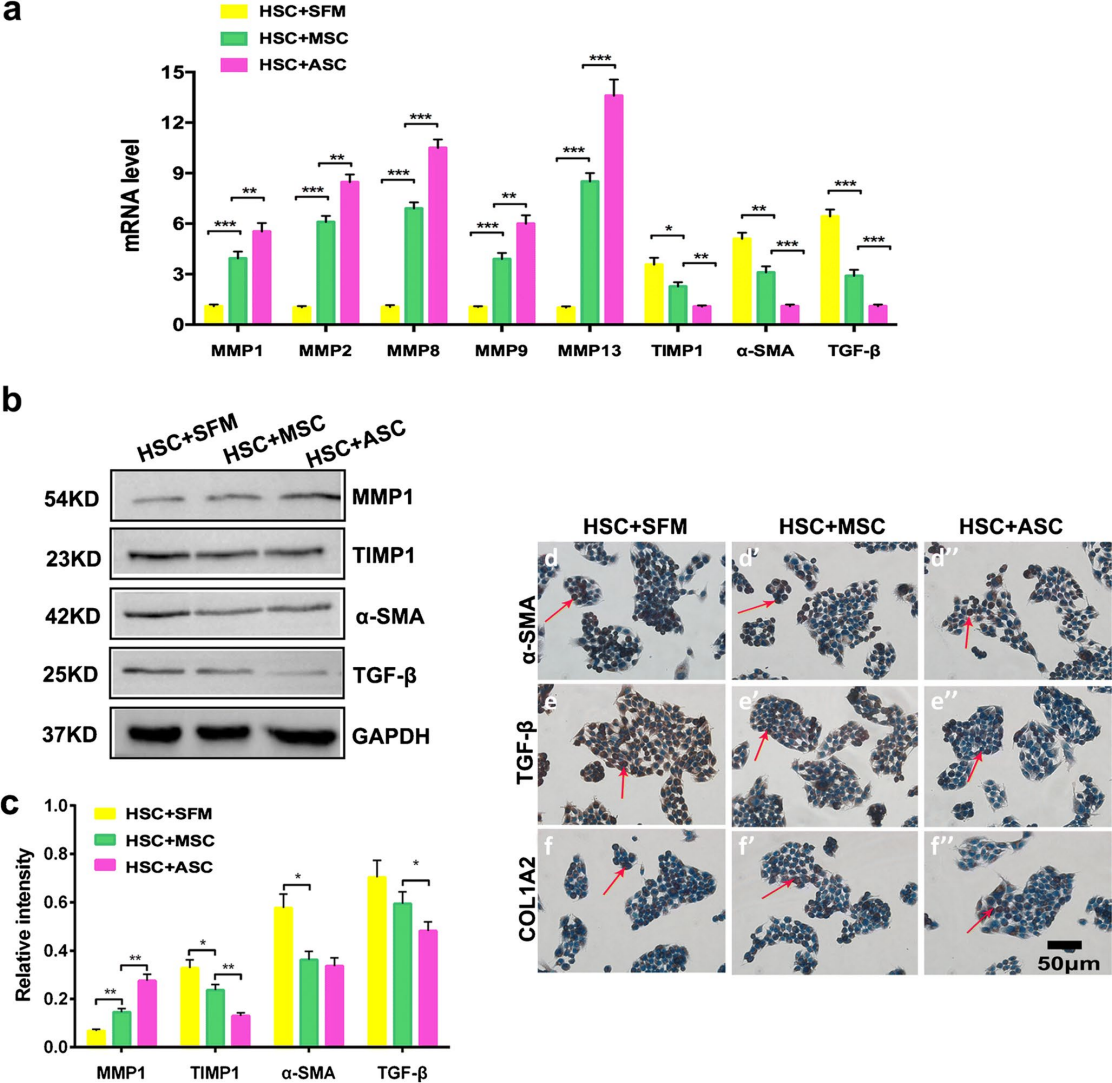
Effects of ASCs on expression of the fibrosis-related genes in HSCs via co-culture approach. a Relative mRNA expression levels of MMPs, TIMP1, α-SMA and TGF-β. b Western blotting analysis of the expression of MMP1, TIMP1, α-SMA and TGF-β. c The relative MMP1, TIMP1, α-SMA and TGF-β intensity via western blotting. Photomicrographs of immunocytochemical staining for α-SMA (d–d″), TGF-β (e–e″) and COL1A2 (f–f″). Note that ASC administration significantly increased the expression of MMPs and decreased expression of TIMP1, α-SMA and TGF-β compared with the CCl_4_ + PBS group; MMPs: matrix metalloproteinases; TIMP1: tissue inhibitors of metalloproteinase 1; α-SMA: anti-α smooth muscle actin; TGF-β: transforming growth factor-β; red arrows indicate positive cells, bar = 50 μm; mean ± SD; *n* = 3; *p < 0.05; **p < 0.01; ***p < 0.001

Next, we detected TGF-β expression using additional methods including qRT-PCR, Western blot and ICC. We found that TGF-β was also decreased in HSCs co-cultured with ASCs, compared to the other two control groups (Fig. 5a–c, e–e″; *p* < 0.05). Moreover, the expression of α-SMA (Fig. 5d″) and COL1A2 (Fig. 5f″′ was decreased in ASCs (compared to the other two control groups, Fig. 5d, d″ and e, e′; *p* < 0.05). Furthermore, the expression of MMP1, MMP2, MMP8, MMP9, and MMP13 was significantly increased, while TIMP1 was decreased compared to the other two control groups (Fig. 5a; *p* < 0.05). Interestingly, the co-culture of ASCs was more effective than MSCs at regulating MMPs, TIMP1, α-SMA, TGF-β and COL1A2 expression in HSCs.

## Discussion

Liver fibrosis is the result of extracellular matrix (ECM) protein deposition, which is mainly mediated by activated HSCs (Elpek 2014; Lee and Friedman 2011). In this study, we investigated the therapeutic effects of ASCs in a CCl_4_-induced rat liver fibrosis model. We found that ASCs administration alleviated liver fibrosis, which includes reducing collagen accumulation, decreasing fatty degeneration, increasing hepatocyte regeneration and significantly enhancing liver functionality. Moreover, ASCs decreased the expression of pro-fibrogenic factors including TGF-β and α-SMA*.* The therapeutic effects were similar to that of the positive control group (MSCs). These results suggested that ASCs transplantation had a beneficial effect on the CCl_4_-induced liver fibrosis model.

More importantly, our study also showed that ASCs inhibit HSC activation and proliferation, controlling the expression of several genes involved in these processes. It has been previously reported that TGF-β regulates the balance between ECM deposition and degradation (Bowen et al. 2013; Sakai et al. 2014). In addition, TGF-β could inhibit the production of collagenase and protease and promote the production of tissue inhibitors of MMPs (Hasan et al. 2016). Here, we also showed that TGF-β was significantly decreased both in CCl_4_ rats and HSCs treated with ASCs. The expression of α-SMA in liver tissue is an indicator of HSC activation, suggesting that activated HSCs expressing α-SMA are involved in the occurrence and development of hepatic fibrosis (Lindert et al. 2005). Here, we demonstrated that α-SMA was significantly decreased both in vivo and vitro. Previous studies have shown that increased MMPs (i.e., MMP-1, -2, -8, -9 and -13) (Nart et al. 2010; Rabani et al. 2010; Zhou et al. 2004) and decreased TIMP1 (Ali et al. 2012) are usually associated with the reversing of fibrosis. Here, we found that the expression of MMPs was increased, while the expression of TIMP1 was decreased in vitro. Previous work indicated that MMP1 promoted HSC apoptosis in the presence of low levels of TIMP1 (Knittel et al. 2000) and that low TIMP1 expression levels promoted the clearance of the fibrotic matrix and reduced the accumulation of ECM (Yoshiji et al. 2002).

**Fig. 6:**
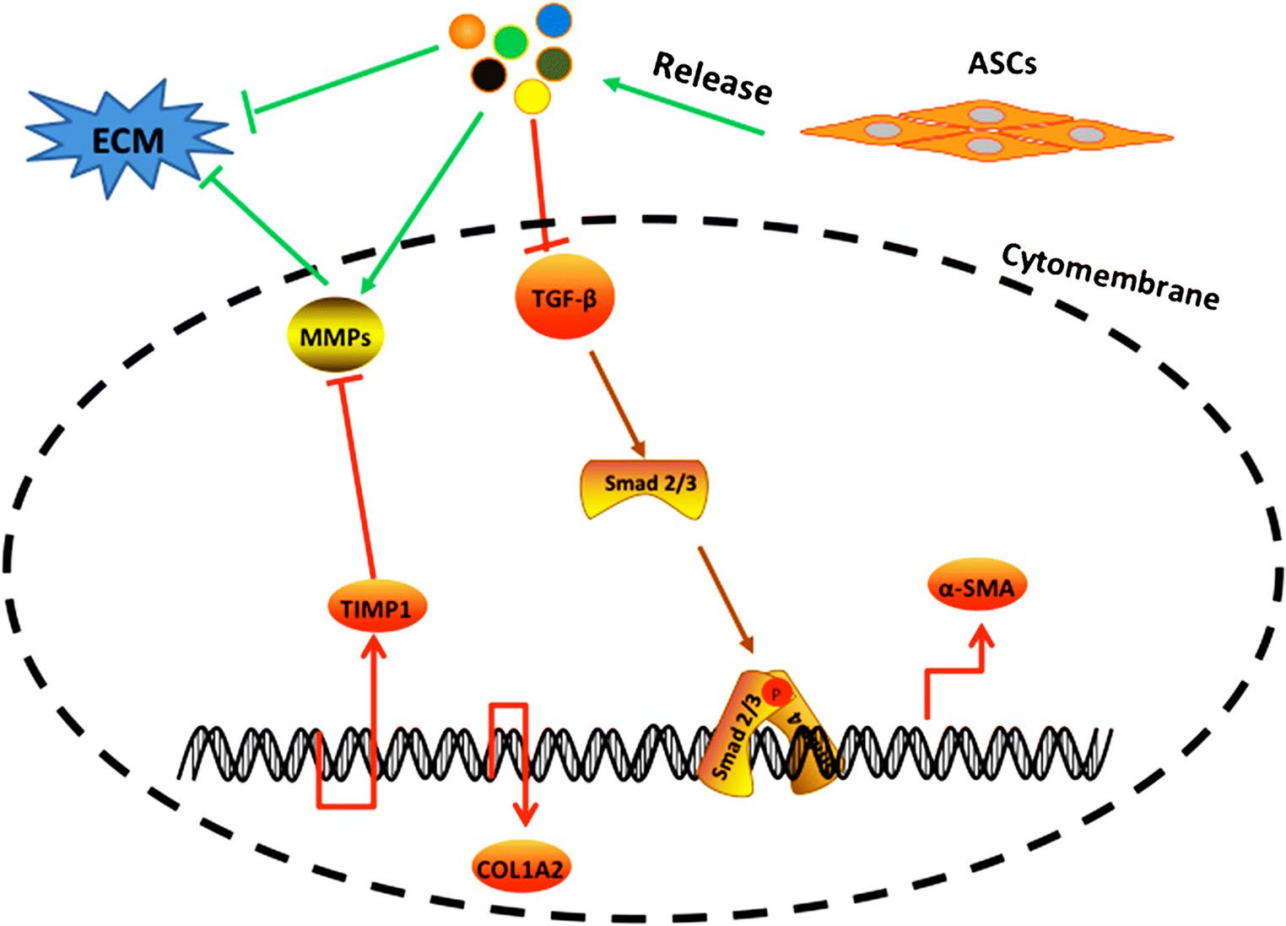
Mechanism underlying reduction of liver fibrosis by ASCs. ASCs releasing factors (assume) reduce ECM and decrease TGF-β expression. ASCs inhibit HSC activation via upregulating the MMP expression and downregulating TIMP1, α-SMA, TGF-β and COL1A2 expression. ASCs: antler stem cells; ECM: extracellular matrix; HSCs: hepatic stellate cells; TGF-β: transforming growth factor-β

The therapeutic properties of MSCs in treating hepatic fibrosis are related to their capacity for hepatocyte-like differentiation (Jiang et al. 2007), trophic factor secretion (Quintanilha et al. 2014), immune-modulatory functions (Aggarwal and Pittenger 2005) and anti-fibrotic activity (Meier et al. 2015). Previous work has shown that MSCs can differentiate into hepatocytes both in vivo and in vitro (Yin et al. 2015). Moreover, MSCs secrete multiple factors that stimulate resident cells to promote the differentiation of native progenitor cells and facilitate recovery of the injured cells (Alfaifi et al. 2018). In fibrotic tissue, MSCs decrease myofibroblast proliferation and promote anti-fibrotic activity (El Agha et al. 2017). At the same time, MSCs are able to reduce the proliferation of activated HSCs and balance ECM synthesis and degradation (Duarte et al. 2015). However, the mechanism by which ASCs may act in the treatment of liver fibrosis is not clear. ASCs may release factors that enhance the level of MMP1, which can degrade the ECM, by inhibiting TIMP1 expression or by direct secretion of MMP1; the other factors that might inhibit HSC activation via inhibiting TGF-β signaling pathway include TGF-β, α-SMA and COL1A2 (Fig. 6).

IL-1, IL-2, IL-6 and TNF-α are the three main inflammatory factors in the inflammation process (Wang et al. 2016). TNF-α is an important factor in the association of inflammatory responses with specific immune responses. It can act on many cell types to induce the production of IL-1 and IL-6 (Wang et al. 2016). These three factors can increase the permeability of capillaries, activate other inflammatory cells and stimulate the formation of oxygen radicals, thus leading to more severe inflammatory responses and damaging intact tissue (Luz-Crawford et al. 2017). In our study, ASC co-culture significantly decreased the upregulation of M1 macrophage gene expression, which typically occurs in response to inflammatory signals (Fig. 4). Thus, we speculated that ASCs promote liver tissue repair by reducing the inflammatory response.

A previous study showed that ASCs have strong proliferation and differentiation ability (Li and Suttie 2011). ASCs can be passaged for dozens of generations while maintaining growth capabilities and in vitro, they can easily differentiate into skin, blood vessels, nerves and other tissues (Li et al. 2010; Li and Suttie 2011). Currently, ASCs have been classified as a special type of MSCs, as they express some key embryonic stem cell markers, such as Oct4, SOX2, Nanog, TERT and nucleostemin (Li and Chu 2016; Li et al. 2009) in addition to classic MSC markers. Although treatment using xenogeneic stem cell transplantation is controversial, a prior study showed that ASCs had no risk of tumor formation and had low immunogenicity (Li and Suttie 2011). In addition, another previous study showed that the characteristics of the complete organ regeneration of deer antlers can be applied to the study of other mammalian organ regeneration (Li et al. 2014), such as liver regeneration. We studied ASCs primarily to find key factors involved and the potential mechanism of liver fibrosis treatment. Our study found that ASCs significantly promote liver regeneration and promote recovery of liver function (Figs. 2 and 3). Therefore, ASCs may be developed into a more efficacious therapeutic reagent than other types of MSCs currently under investigation for liver fibrosis in the clinic. In the future studies, we will use the ASC exosomes for treating liver fibrosis to overcome the problem of immunocompatibility thus leading to the development of effective and safe cell-free regenerative reagents with predictable therapeutic effects.

## Conclusion

In conclusion, our study showed that ASCs from the antler growth center alleviate liver fibrosis, inhibit HSC activation, promote hepatocyte regeneration, decrease inflammation and restore liver function. These results are extremely promising and suggest that ASCs may be used as a novel stem cell source for the treatment of liver fibrosis in the clinic. Understanding ASCs and the relevant molecules that regulate liver regeneration may provide a new therapeutic approach for reducing liver fibrosis.

## Electronic supplementary material

ESM 1 (DOCX 130 kb)

## References

[CR1] Aggarwal S, Pittenger MF (2005) Human mesenchymal stem cells modulate allogeneic immune cell responses. Blood 105:1815–1822 15494428 10.1182/blood-2004-04-1559

[CR2] Albanis E, Friedman SL (2001) Hepatic fibrosis. Pathogenesis and principles of therapy. Clin Liver Dis 5:315–334 v-vi 11385966 10.1016/s1089-3261(05)70168-9

[CR3] Alfaifi M, Eom YW, Newsome PN, Baik SK (2018) Mesenchymal stromal cell therapy for liver diseases. J Hepatol 68:1272–1285 29425678 10.1016/j.jhep.2018.01.030

[CR4] Ali G, Mohsin S, Khan M, Nasir GA, Shams S, Khan SN, Riazuddin S (2012) Nitric oxide augments mesenchymal stem cell ability to repair liver fibrosis. J Transl Med 10:75 22533821 10.1186/1479-5876-10-75 PMC3419634

[CR5] Bataller R, Brenner DA (2005) Liver fibrosis. J Clin Investig 115:209–218 15690074 10.1172/JCI24282 PMC546435

[CR6] Bowen T, Jenkins RH, Fraser DJ (2013) MicroRNAs, transforming growth factor beta-1, and tissue fibrosis. J Pathol 229:274–285 23042530 10.1002/path.4119

[CR7] Cho KA, Woo SY, Seoh JY, Han HS, Ryu KH (2012) Mesenchymal stem cells restore CCl4-induced liver injury by an antioxidative process. Cell Biol Int 36:1267–1274 23035905 10.1042/CBI20110634

[CR8] Duarte S, Baber J, Fujii T, Coito AJ (2015) Matrix metalloproteinases in liver injury, repair and fibrosis. Matrix Biol 44-46:147–156 25599939 10.1016/j.matbio.2015.01.004 PMC4495728

[CR9] El Agha E, Kramann R, Schneider RK, Li X, Seeger W, Humphreys BD, Bellusci S (2017) Mesenchymal stem cells in fibrotic disease. Cell Stem Cell 21:166–177 28777943 10.1016/j.stem.2017.07.011

[CR10] Elpek GO (2014) Cellular and molecular mechanisms in the pathogenesis of liver fibrosis: an update. World J Gastroenterol 20:7260–7276 24966597 10.3748/wjg.v20.i23.7260 PMC4064072

[CR11] Eom YW, Shim KY, Baik SK (2015) Mesenchymal stem cell therapy for liver fibrosis. Korean J Intern Med 30:580–589 26354051 10.3904/kjim.2015.30.5.580 PMC4578027

[CR12] Hasan IH, El-Desouky MA, Hozayen WG, Abd el Aziz GM (2016) Protective effect of Zingiber Officinale against CCl4-induced liver fibrosis is mediated through downregulating the TGF-beta1/Smad3 and NF-kB/IkB pathways. Pharmacology 97:1–9 26551763 10.1159/000441229

[CR13] Huang B, Cheng X, Wang H, Huang W, la Ga HZ, Wang D, Zhang K, Zhang H, Xue Z, Da Y, Zhang N, Hu Y, Yao Z, Qiao L, Gao F, Zhang R (2016) Mesenchymal stem cells and their secreted molecules predominantly ameliorate fulminant hepatic failure and chronic liver fibrosis in mice respectively. J Transl Med 14:45 26861623 10.1186/s12967-016-0792-1 PMC4746907

[CR14] Jiang ZS, Gao Y, Mu N (2007) Multipotent adult progenitor cells from human bone marrow differentiate into hepatocyte-like cells induced by co-culture with human hepatocyte line. Zhonghua Yi Xue Za Zhi 87:414–418 17456385

[CR15] Kisseleva T, Brenner DA (2012) The phenotypic fate and functional role for bone marrow-derived stem cells in liver fibrosis. J Hepatol 56:965–972 22173163 10.1016/j.jhep.2011.09.021 PMC3307836

[CR16] Knittel T, Mehde M, Grundmann A, Saile B, Scharf JG, Ramadori G (2000) Expression of matrix metalloproteinases and their inhibitors during hepatic tissue repair in the rat. Histochem Cell Biol 113:443–453 10933221 10.1007/s004180000150

[CR17] Lan L, Liu R, Qin LY, Cheng P, Liu BW, Zhang BY, Ding SZ, Li XL (2018) Transplantation of bone marrow-derived endothelial progenitor cells and hepatocyte stem cells from liver fibrosis rats ameliorates liver fibrosis. World J Gastroenterol 24:237–247 29375209 10.3748/wjg.v24.i2.237 PMC5768942

[CR18] Lee UE, Friedman SL (2011) Mechanisms of hepatic fibrogenesis. Best Pract Res Clin Gastroenterol 25:195–206 21497738 10.1016/j.bpg.2011.02.005 PMC3079877

[CR19] Li C, Chu W (2016) The regenerating antler blastema: the derivative of stem cells resident in a pedicle stump. Front Biosci (Landmark edition) 21:455–467 10.2741/4401 26709786

[CR20] Li C, Suttie JM (2003) Tissue collection methods for antler research. Eur J Morphol 41:23–30 15121545 10.1076/ejom.41.1.23.28106

[CR21] Li C, Yang F, Haines S, Zhao H, Wang W, Xing X, Sun H, Chu W, Lu X, Liu L, McMahon C (2010) Stem cells responsible for deer antler regeneration are unable to recapitulate the process of first antler development-revealed through intradermal and subcutaneous tissue transplantation. J Exp Zool B Mol Dev Evol 314:552–570 20549758 10.1002/jez.b.21361

[CR22] Li C, Yang F, Sheppard A (2009) Adult stem cells and mammalian epimorphic regeneration-insights from studying annual renewal of deer antlers. Current stem cell research & therapy 4:237–251 19492976 10.2174/157488809789057446

[CR23] Li CYF, Suttie JM (2011) Stem cells, stem cell niche and antler development. Anim Prod Sci 51:267–276

[CR24] Li C, Zhao H, Liu Z, McMahon C (2014) Deer antler--a novel model for studying organ regeneration in mammals. Int J Biochem Cell Biol 56:111–122 25046387 10.1016/j.biocel.2014.07.007

[CR25] Lindert S, Wickert L, Sawitza I, Wiercinska E, Gressner AM, Dooley S, Breitkopf K (2005) Transdifferentiation-dependent expression of alpha-SMA in hepatic stellate cells does not involve TGF-beta pathways leading to coinduction of collagen type I and thrombospondin-2. Matrix Biol 24:198–207 15905080 10.1016/j.matbio.2005.03.003

[CR26] Luz-Crawford P, Jorgensen C, Djouad F (2017) Mesenchymal stem cells direct the immunological fate of macrophages. Results Probl Cell Differ 62:61–72 28455706 10.1007/978-3-319-54090-0_4

[CR27] Meier RP, Mahou R, Morel P, Meyer J, Montanari E, Muller YD, Christofilopoulos P, Wandrey C, Gonelle-Gispert C, Buhler LH (2015) Microencapsulated human mesenchymal stem cells decrease liver fibrosis in mice. J Hepatol 62:634–641 25450712 10.1016/j.jhep.2014.10.030

[CR28] Nart D, Yaman B, Yilmaz F, Zeytunlu M, Karasu Z, Kilic M (2010) Expression of matrix metalloproteinase-9 in predicting prognosis of hepatocellular carcinoma after liver transplantation. Liver Transplant : official publication of the American Association for the Study of Liver Diseases and the International Liver Transplantation Society 16:621–630 10.1002/lt.22028 20440771

[CR29] Nishikawa H, Osaki Y (2015) Liver cirrhosis: evaluation, nutritional status, and prognosis. Mediat Inflamm 2015:872152 10.1155/2015/872152 PMC4606163 26494949

[CR30] Quintanilha LF, Takami T, Hirose Y, Fujisawa K, Murata Y, Yamamoto N, Goldenberg RC, Terai S, Sakaida I (2014) Canine mesenchymal stem cells show antioxidant properties against thioacetamide-induced liver injury in vitro and in vivo. Hepatol Res 44:E206–E217 23889977 10.1111/hepr.12204

[CR31] Rabani V, Shahsavani M, Gharavi M, Piryaei A, Azhdari Z, Baharvand H (2010) Mesenchymal stem cell infusion therapy in a carbon tetrachloride-induced liver fibrosis model affects matrix metalloproteinase expression. Cell Biol Int 34:601–605 20178458 10.1042/CBI20090386

[CR32] Sakai K, Jawaid S, Sasaki T, Bou-Gharios G, Sakai T (2014) Transforming growth factor-beta-independent role of connective tissue growth factor in the development of liver fibrosis. Am J Pathol 184:2611–2617 25108224 10.1016/j.ajpath.2014.06.009 PMC4715221

[CR33] Sun H, Yang F, Chu W, Zhao H, McMahon C, Li C (2012) Lentiviral-mediated RNAi knockdown of Cbfa1 gene inhibits endochondral ossification of antler stem cells in micromass culture. PLoS One 7:e47367 23056636 10.1371/journal.pone.0047367 PMC3467256

[CR34] Wang LT, Ting CH, Yen ML, Liu KJ, Sytwu HK, Wu KK, Yen BL (2016) Human mesenchymal stem cells (MSCs) for treatment towards immune- and inflammation-mediated diseases: review of current clinical trials. J Biomed Sci 23:76 27809910 10.1186/s12929-016-0289-5 PMC5095977

[CR35] Watanabe Y, Tsuchiya A, Seino S, Kawata Y, Kojima Y, Ikarashi S, Starkey Lewis PJ, Lu WY, Kikuta J, Kawai H, Yamagiwa S, Forbes SJ, Ishii M, Terai S (2019) Mesenchymal stem cells and induced bone marrow-derived macrophages synergistically improve liver fibrosis in mice. Stem Cells Transl Med 8:271–284 30394698 10.1002/sctm.18-0105 PMC6392382

[CR36] Wu X, Wu X, Ma Y, Shao F, Tan Y, Tan T, Gu L, Zhou Y, Sun B, Sun Y, Wu X, Xu Q (2016) CUG-binding protein 1 regulates HSC activation and liver fibrogenesis. Nat Commun 7:13498 27853137 10.1038/ncomms13498 PMC5118555

[CR37] Yin L, Zhu Y, Yang J, Ni Y, Zhou Z, Chen Y, Wen L (2015) Adipose tissue-derived mesenchymal stem cells differentiated into hepatocyte-like cells in vivo and in vitro. Mol Med Rep 11:1722–1732 25395242 10.3892/mmr.2014.2935 PMC4270341

[CR38] Yoshiji H, Kuriyama S, Yoshii J, Ikenaka Y, Noguchi R, Nakatani T, Tsujinoue H, Yanase K, Namisaki T, Imazu H, Fukui H (2002) Tissue inhibitor of metalloproteinases-1 attenuates spontaneous liver fibrosis resolution in the transgenic mouse. Hepatology (Baltimore, Md) 36:850–860 12297832 10.1053/jhep.2002.35625

[CR39] Zhou X, Hovell CJ, Pawley S, Hutchings MI, Arthur MJ, Iredale JP, Benyon RC (2004) Expression of matrix metalloproteinase-2 and -14 persists during early resolution of experimental liver fibrosis and might contribute to fibrolysis. Liver Int : official journal of the International Association for the Study of the Liver 24:492–501 10.1111/j.1478-3231.2004.0946.x 15482348

