# Correction to: Antler stem cells as a novel stem cell source for reducing liver fibrosis

**DOI:** 10.1007/s00441-025-04023-8

**Published:** 2025-11-28

**Authors:** Xiaoli Rong, Yanyan Yang, Guokun Zhang, Haiying Zhang, Chunyi Li, Yimin Wang

**Affiliations:** 1 The Scientific Research Center, China-Japan Union Hospital of Jilin University, 126 Xiantai St., Changchun, 130033 Jilin China; 2 Department of Ultrasound, China-Japan Union Hospital of Jilin University, 126 Xiantai St., Changchun, 130033 Jilin China; 3 Institute of Special Wild Economic Animals and Plants, Chinese Academy of Agricultural Sciences, 4899 Juye St., Changchun, 130112 Jilin China; 4 Key Laboratory of Pathobiology, Ministry of Education, Norman Bethune College of Medicine, Jilin University, 126 Xinmin St., Changchun, 130021 Jilin China

**Correction to: Cell and Tissue Research (2020) 379:195–206**

10.1007/s00441-019-03081-z

The authors corrected the image that the version of Figure 1d’’ that appeared in original published article is incorrect.

The correct version appears below.

**Figure:**
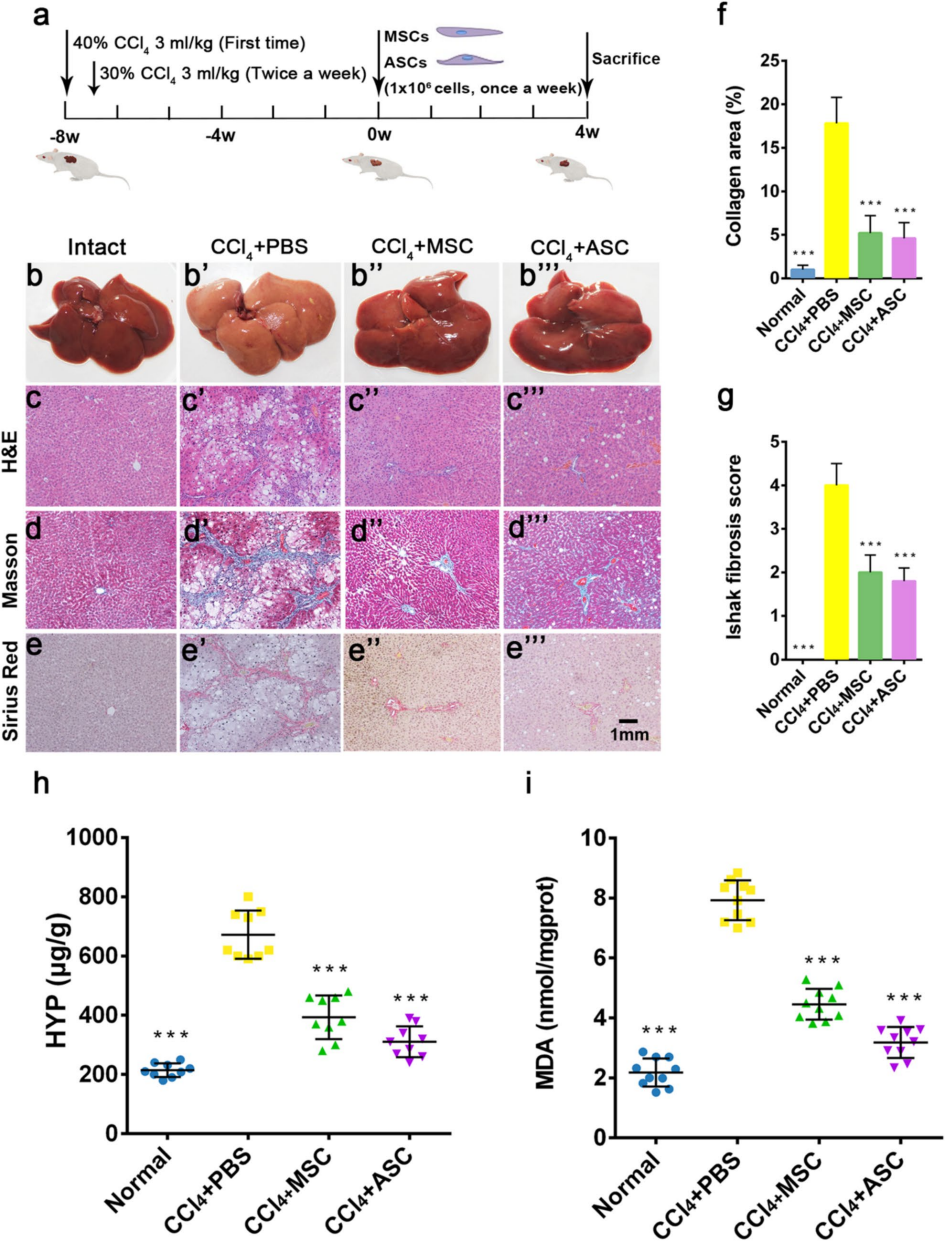
**Fig. 1** Effects of ASCs on liver fibrosis in CCl4 treated rats. a Experimental design. Liver (a–a″′) and liver tissue section, stained with H&E (c–c″′), Masson (b–b″′) and Sirius Red (e–e″′), bar = 1 mm. f Collagen abundance in the livers assessed by area quantification using computer-assisted image analysis. g Histopathological analysis of liver sections via Ishak scoring criteria. Ishak score from 0 to 6 (0 = no fibrosis, 6 = cirrhosis): mild (Ishak, 0–2) to severe fibrosis (Ishak, 3–6). h HYP levels. i MDA levels. Note that ASC administration significantly alleviated the liver fibrosis compared with the CCl4 + PBS group; no significant difference to the intact group at levels of HYP and MDA; and there was a strong trend for ASC that had a better effect on reducing liver fibrosis than MSC, although statistically not significant. ASCs: antler stem cells; MSCs: mesenchymal stem cells; HYP: hydroxyproline; MDA: malondialdehyde; mean ± SD; n = 10. ***p < 0.001

The incorrect version appears below.

**Figure:**
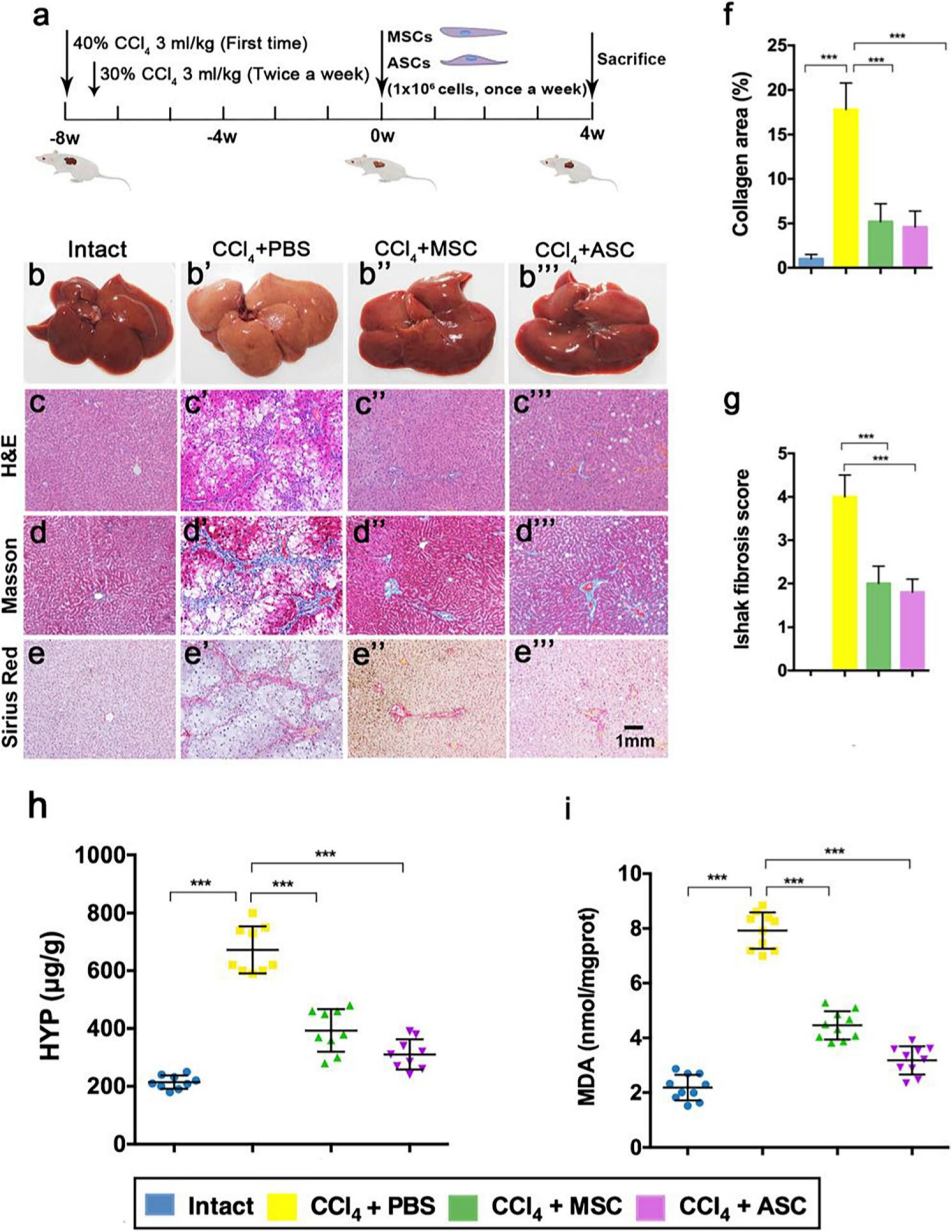
**Fig. 1** Effects of ASCs on liver fibrosis in CCl4 treated rats. a Experimental design. Liver (a–a″′) and liver tissue section, stained with H&E (c–c″′), Masson (b–b″′) and Sirius Red (e–e″′), bar = 1 mm. f Collagen abundance in the livers assessed by area quantification using computer-assisted image analysis. g Histopathological analysis of liver sections via Ishak scoring criteria. Ishak score from 0 to 6 (0 = no fibrosis, 6 = cirrhosis): mild (Ishak, 0–2) to severe fibrosis (Ishak, 3–6). h HYP levels. i MDA levels. Note that ASC administration significantly alleviated the liver fibrosis compared with the CCl4 + PBS group; no significant difference to the intact group at levels of HYP and MDA; and there was a strong trend for ASC that had a better effect on reducing liver fibrosis than MSC, although statistically not significant. ASCs: antler stem cells; MSCs: mesenchymal stem cells; HYP: hydroxyproline; MDA: malondialdehyde; mean ± SD; n = 10. ***p < 0.001

The original article has been corrected.

