# Supplementary material for: Antler stem cells as a novel stem cell source for reducing liver fibrosis
Source: Cell Tissue Res. 2019 Aug 19;379(1):195–206. doi: 10.1007/s00441-019-03081-z (PMC12660448; doi:10.1007/s00441-019-03081-z)
Supplement: Supplementary file 1 — (DOCX 130 kb) [file 441_2019_3081_MOESM1_ESM.docx]

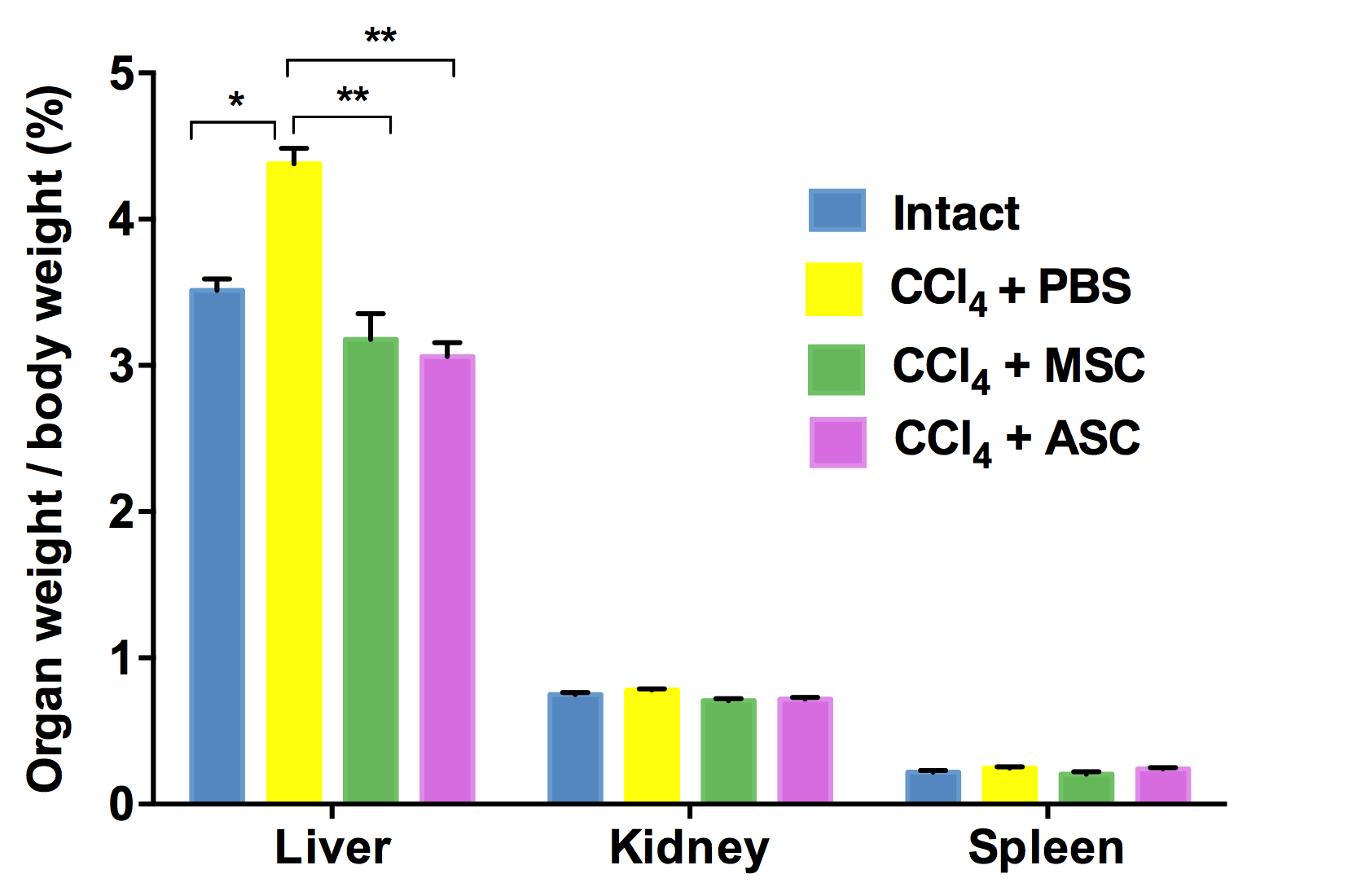


**Fig. S1** The ratio of organ weight (liver, kidney and spleen) to body weight in CCl4-induced liver fibrosis rats. **p*<0.05, ***p*<0.01, n=10.
